# Validity and Reliability of the Dyslexia Checklist for Chinese Children

**DOI:** 10.3389/fpsyg.2018.01915

**Published:** 2018-10-09

**Authors:** Fang Hou, Ling Qi, Lingfei Liu, Xiu Luo, HuaiTing Gu, Xinyan Xie, Xin Li, Jiajia Zhang, Ranran Song

**Affiliations:** ^1^Department of Maternal and Child Health, MOE Key Laboratory of Environment and Health, School of Public Health, Tongji Medical College, Huazhong University of Science and Technology, Wuhan, China; ^2^School of Health Science and Nursing, Wuhan Polytechnic University, Wuhan, China; ^3^Department of Epidemiology and Biostatistics, Arnold School of Public Health, University of South Carolina, Columbia, SC, United States

**Keywords:** dyslexia, screen, validity, reliability, reading skills

## Abstract

The study on developmental dyslexia (DD) has fairly matured in the past decades, even when there is a lack of a standardized and convenient instrument for dyslexia in the Chinese population. The purpose of this study was to assess the reliability and validity of the Dyslexia Checklist for Chinese Children (DCCC), which was administered to Chinese students in primary school. A total of 545 students from grades 2 through 6 were recruited in Wuhan to participate in this study. We used confirmatory factor analysis (CFA) to evaluate the structure validity of the DCCC. Concurrent validity was determined via correlations between the DCCC and the verbal comprehension index (VCI), and Chinese achievement. The reliability of the DCCC was assessed via test-retest reliability and internal consistency. The CFA suggested that the first order model with eight factors and 55 items fit the data well (RMSEA = 0.057, CFI = 0.930, and TLI = 0.925). The DCCC was negatively associated with VCI (*r* = −0.218) and Chinese achievement (*r* = −0.372). The test-retest reliability of the DCCC was 0.734, and the internal consistency of all subscales was above 0.752. The DCCC thus proved to have adequate validity and reliability to screen Chinese dyslexia among students in grades 2 through 6.

## Introduction

Developmental dyslexia (DD) is a specific learning disorder, occurring in all languages. According to the definition by the International Dyslexia Association (IDA), dyslexia is marked by impairment in accurate and/or fluent word recognition, spelling and decoding abilities. Even though children with DD have no difference in education and sociocultural resources than typically developed children, their reading abilities are below the levels expected for their current ages (Becker et al., 2017). The consequence is that children with DD will have problems in reading comprehension and social skills that may be adverse to their academic achievements and incomes in adulthood (McLaughlin et al., 2014; Ghisi et al., 2016). Thus, it is important to screen the children who are at the risk of DD as early and as possible, and then the targeted interventions can be implemented timely.

There is not a consistent tool, such as the Wechsler Intelligence Scale for Intelligence Quotient, to identify dyslexia. According to the Diagnostic and statistical Manual of Mental Disorders (DSM), the core symptoms of dyslexia are impairment in word-reading accuracy, reading rate and fluency, and reading comprehension. Based on these core symptoms, a number of foreign reading-related tests from different instruments were used to evaluate reading skill (Snowling et al., 2012; Di Liberto et al., 2018; Moreau et al., 2018). And also there are several tests used to identify children with dyslexia in the Chinese mainland (Yang and Gong, 1997; Yang et al., 2017), but these tests mainly focus on the amount of recognized characters. This is not consistent with the current theories that dyslexia is not an ‘all-or-none’ condition but rather multi-deficits in a range of perceptual and cognitive processes (Snowling et al., 2012; Ozernov-Palchik et al., 2017). Another important limitation is that the reading tests might be too complex and difficult for standardization, and it is not convenient to apply these tests to a large sample. On the other hand, Chinese differs in its written orthography, which is different from alphabetic languages (e.g., English and Finnish). The Chinese characters are made up of different stroke patterns (e.g., 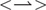 and 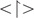) rather than alphabet letters (e.g., a, b, and c) (Chung et al., 2018). Evidence suggested that phonological awareness is significant for reading skill in Chinese just as in alphabetic orthographies. However, in contrast to alphabetic languages in which awareness of phonemes is critically important, morphological and syllabic awareness play a large role in learning to read Chinese (Peterson and Pennington, 2015). Therefore, matured foreign screening instruments could not be applied to screen dyslexia among Chinese students. In order to screen dyslexic children in a time-saving way in China, which has the largest population in the world, Wu established The Dyslexia Checklist for Chinese Children (DCCC) in 2006 (Wu et al., 2006b).

The DCCC is a parent-report scale designed to measure the reading ability of Chinese students in grades 3 through 6 in the Chinese mainland. The initial scale contains 57 items that were based on the definition of dyslexia in ICD-10, DSM-IV, and clinical symptoms described in relative references (Liu et al., 2016). Among these 57 items, 55 items have loading on eight factors including the deficit of vocabulary comprehension, the visual deficit of word recognition, the auditory deficit of word recognition, the deficit of spelling, the deficit of written expression and attention, the deficit of oral language, and bad reading habits. The remaining two items are the family risk of dyslexia and mathematic ability, respectively, which were considered as **Supplementary Material** (Wu et al., 2006b). Compared to the individual interview, the parent-reported scale can provide more information about the children, such as classic symptoms of dyslexia, family history, and so on. For identification of neurodevelopmental disorders such as Attention Deficit Hyperactivity Disorder and Autism Spectrum Disorder, parent-reported scales were widely used. However, the DCCC is a preliminary screening scale. The identification of dyslexia is accomplished via combining the DCCC, academic achievement (below the tenth percentile of all children in the same grade), and the Chinese edition of “The Pupil Rating Scale Revised—Screening for Learning Disabilities (the score lower than 65).” Our team used this approach to explore and report the prevalence and risk factors of dyslexia in the Chinese mainland (Sun et al., 2013; Kong et al., 2016; Shao et al., 2016).

The cognition of the individual can be affected by culture, economy, society, and so on. As time goes by, psychological instruments need to be revised. For example, the fourth edition of Wechsler Intelligence Scale for Children (WISC) was established in 2003, and then the WISC-V was published in 2014. Twelve years passed since the introduction of the DCCC, the economy of China developed rapidly, and the life of the Chinese has become better and happier. There is a need to revalidate the usage of the DCCC and the necessaries in revising or rewording to adapt it to the students in different educational environments. For the dyslexia definition, DSM-IV proposed that there were different nosological categories of learning disabilities (arithmetical, reading, spelling), while in DSM-V there is only Specific Learning Disorder and reinstated the term of dyslexia instead of Learning Disorder (Tannock, 2013). Because the core symptoms were not changed, we did not delete or add items to the current DCCC and want to assess that it is still a reliable instrument to identify dyslexia now.

Previous studies indicated the Full Scale Intelligence Quotient negatively associated with the score of the DCCC, and the Chinese achievement not only directly reflected the reading performance but also related with intelligence (Wu et al., 2006a; Lopes-Silva et al., 2016). Thus, we hypothesize that the verbal comprehension index (VCI) of WISC and the *Z*-scores of Chinese achievement would be negatively associated with the eight subscale scores and the total overall score of the DCCC.

The importance of early identification of DD is widely acknowledged (Snowling, 2013). Early identification can allow early intervention to be implemented to mitigate some of the negative effects associated with reading skills (Poulsen et al., 2017). Thus, universal screening instruments can be applied to pupils as early as the beginning of primary school and have been revised constantly to apply to younger children. For example, the Kaufman Test of Educational Achievement, second edition, can be used to identify dyslexia among individuals aged from 4 years and 6 months to 25 years and 11 months (Staff, 2008), while the third edition can be applied to individuals aged from 4 to 26 years (Parkin and Frisby, 2018). For the DCCC, there was no doubt that the greatest limitation was the beginning age for identifying dyslexia. The scale was established for students in grades 3 through 6, while most instruments can be applied to pupils and even preschool children. So, we want to assess whether DCCC can be used among children in grade 2.

In this study, we first assess the DCCC is still a reliable instrument to identify dyslexia among students in grades 3–6, and then show the DCCC can be applied to students in grade 2.

## Materials and Methods

### Participants and Procedure

We recruited 616 students in grades 2 through 6 from a common primary school in Wuhan, China. Inclusion criteria were: (1) native Chinese Han students, (2) no visual and auditory disorders, learning disorders or psychiatric diseases, as reported by parents, and (3) and normal IQ. The parents of eligible students were given written informed consent and the questionnaire; 557 parents voluntarily completed their questionnaire and written informed consent, and then the researchers excluded the unqualified questionnaires, for which the completion of the DCCC was lower than 90%. Thus, the final sample size was 545. If the parents allowed their children to continue to participate in other test, trained researchers would conduct verbal comprehension subscales of WISC-CR on the eligible students (*n* = 352). Two weeks after the first questionnaire completed, a following new questionnaire was sent to the parents who agree to complete the questionnaire a second time (*n* = 133).

Demographic data, including age, sex, paternal ages, educational level, and incomes were collected by the questionnaire. Teachers further provided the score of Chinese achievement after the midterm exam was finished. The *Z*-score of Chinese achievement for the grade was obtained using the *Z*-score formula. The Ethics Committee of Tongji Medical College, Huazhong University of Science and Technology, China approved this study.

### Instruments

#### Dyslexia Checklist for Chinese Children (DCCC)

The DCCC was established to screen Chinese dyslexia among the students in grades 3 through 6. This is a parents-report scale including 55 items loading on eight factors. The score of each item ranged from 1 to 5 (1, never; 2, rarely; 3, sometimes; 4, often; and 5, always), and the highest score represents the worst reading ability.

#### Wechsler Intelligence Scale for Children (WISC)-IV Chinese Version

The WISC is an instrument used worldwide to assess the intelligence among children aged from 6 to 16. Zhang (2009) revised the Chinese version of WISC-IV. The full scale IQ provided information of general intelligence, while four index scores, including VCI, Perceptual Reasoning Index, Working Memory Index, and Processing Speed Index, represented specific cognitive ability. In this study, the researchers with certificates of WISC-CR only conducted verbal comprehension subscale on the students whose parents completed the DCCC and agreed to participate in the test. The Verbal Comprehension subscale is comprised of three core tests, including Similarities, Vocabulary, and Comprehension (Thaler et al., 2015). The higher score means the higher verbal-performance.

### Data Analysis

Confirmatory factor analysis (CFA) was used to test the overall fit of the data to the scale model with 55 items and 8 factors. Weighted least squares with mean and variance adjustment (WLSMV) were used in this CFA. Indices used to evaluate the model fit include the comparative fit index (CFI), the Tuckere Lewis Index (TLI), root mean square error of approximation (RMSEA), weighted root mean square of residual (WRMR), and χ^2^/df. The index criteria for well-fitting models were: CFI > 0.90, GFI > 0.90, RMSEA < 0.08, and 2 < χ^2^/df < 5 (Lance et al., 2006). The value of WRMR closer to 1.0 indicates an acceptable fit (Daundasekara et al., 2017). According to Kline, the items with lower factor loading (<0.35) would be removed (Quah et al., 2017).

Concurrent validity was assessed via associations between the score of the DCCC, the VCI, and the *Z*-score of Chinese achievement for grade. According to previous studies, we hypothesized that the DCCC scores might be negatively related to the VCI and Chinese achievement.

The reliability of the DCCC was determined by the test-retest reliability and internal consistency. Test-retest reliability was estimated by intra class coefficient (ICC), which was calculated by the correlation between the first and second completion of the scale. An adequate value above 0.60 and 0.70 for ICC is better (Aleksic et al., 2017). Cronbach’s alpha was used to determine internal consistency of the scale. An acceptable cutoff value is 0.70 (Lance et al., 2006). The CFA was performed using Mplus, version 7.0, and SPSS, version 19.0 was used for other statistics analysis.

## Results

### Demographic Characteristics

**Table 1** shows the demographics of the sample. The students’ sample comprised 545 subjects (273 boys, 257 girls, missing 15; grades range: 2–6). Mean age of the students’ parents was 38.50 ± 4.51 years for fathers and 36.35 ± 4.39 years for mothers, respectively. Concerning educational attainment for parents, 16.94% (for father) and 11.94% (for mother) had undergraduate degrees.

**Table 1:** Shows the demographic information of participants, including sex, grade, and household, and so on.

Characteristics		*n*	Percentage (%)
Sex	Boys	273	51.51
	Girls	257	48.49
Grade	2	91	16.70
	3	97	17.80
	4	89	16.33
	5	116	21.28
	6	152	27.89
Family income	<¥50,000	108	27.91
	> = ¥50,000	279	72.09
Age of father	<30	11	2.59
	30–40	304	71.70
	> = 40	109	25.71
Age of mother	<30	30	7.04
	30–40	333	78.17
	> = 40	63	14.79
Education level of father	Junior high school diploma or below	194	45.65
	High school diploma	159	37.41
	Undergraduate degree	72	16.94
Education level of mother	Junior high school diploma or below	221	51.76
	High school diploma	155	36.30
	Undergraduate degree	51	11.94

### Confirmatory Factor Analyses (CFA) of the DCCC

Confirmatory factor analysis was performed among the students in grades 3–6 to test the fit of eight models regarding the factor structure of the DCCC. Due to categorical variables and violation of the assumption of multivariate normality, model fit was estimated with the robust weighted least squares method (MLSMV). The first CFA model represented the 55-item, 8-factor structure that the DCCC recently proposed, in an attempt to determine whether any items might be inappropriate for the set of questions suggested. The first model showed RMSEA of 0.057, CFI of 0.930, TLI of 0.925, and WRMR of 1.445, and the value of χ^2^/df was 2.464. According to Lance et al. (2006), the indices suggested a good fit for the model and thus confirmed the eight-factor structure. Furthermore, the factor loading for the first model was used to identify whether items load strongly onto their hypothesized latent factor. According to Kline’s criterion, the items with a factor loading below 0.35 should be removed. Item 5 was removed from the scale due to a poor factor loading (0.143). The resulting model showed a good fit to the data (RMSEA = 0.058, CFI = 0.929, TLI = 0.924, and WRMR = 1.456). The index stayed similar. The modification indices suggested there are no strong correlations among all items, so we still used the first model, including 55 items and 8 factors. The standardized factor loadings of CFA are shown in **Table 2**. Additionally, we also performed CFA among the students in grade 2. The results showed a good fit to the data (RMSEA = 0.052, CFI = 0.956, TLI = 0.953, and WRMR = 0.991).

**Table 2:** Shows the standardized factor loadings of the confirmatory factor analysis.

	Sub1	Sub2	Sub3	Sub4	Sub5	Sub6	Sub7	Sub8
DCCC15	0.383							
DCCC27	0.698							
DCCC30	0.754							
DCCC36	0.701							
DCCC38	0.777							
DCCC40	0.626							
DCCC19		0.682						
DCCC31		0.692						
DCCC35		0.725						
DCCC41		0.620						
DCCC52		0.808						
DCCC53		0.768						
DCCC57		0.558						
DCCC3			0.515					
DCCC37			0.801					
DCCC42			0.671					
DCCC43			0.727					
DCCC47			0.514					
DCCC49			0.729					
DCCC4				0.666				
DCCC14				0.611				
DCCC18				0.625				
DCCC24				0.728				
DCCC25				0.848				
DCCC34				0.681				
DCCC45				0.556				
DCCC1					0.645			
DCCC2					0.685			
DCCC5					0.143			
DCCC6					0.735			
DCCC7					0.661			
DCCC22					0.679			
DCCC55					0.856			
DCCC9						0.663		
DCCC16						0.716		
DCCC20						0.620		
DCCC28						0.768		
DCCC39						0.679		
DCCC48						0.793		
DCCC54						0.809		
DCCC8							0.724	
DCCC11							0.687	
DCCC17							0.617	
DCCC21							0.772	
DCCC23							0.787	
DCCC56							0.713	
DCCC10								0.769
DCCC26								0.700
DCCC29								0.695
DCCC32								0.629
DCCC33								0.805
DCCC44								0.420
DCCC46								0.800
DCCC50								0.712
DCCC51								0.631

*Sub1 = DCCC factor 1…Sub8 = DCCC factor 8.*

### Reliability Statistics

**Table 3** presents the internal consistency (Cronbach’s α) indices and test-retest correlations for the DCCC and each factor among students in grades 3–6 and 2, respectively. For the students in grades in 3 to 6, the internal consistency for the total score on the DCCC was 0.974, and the internal consistency for the factors ranged from 0.752 to 0.901. The above results indicated good internal consistency of this scale. To assess the reliability and reproducibility of the instrument, retest was performed on 109 subjects who received the DCCC a second time. The test-retest reliability was good to excellent for the total score of the DCCC (0.734) and each factor (Sub1 = 0.647, Sub2 = 0.706, Sub3 = 0.690, Sub4 = 0.637, Sub5 = 0.615, Sub6 = 0.736, Sub7 = 0.689, and Sub8 = 0.662). Among students in grade 2, the internal consistency for the factors was above 0.718, and the test-retest reliability ranged from 0.537 to 0.770. All results of test-retest correlations and internal consistency indicated that the DCCC is a reliable instrument to identify dyslexia for students from grades 2 through 6.

**Table 3:** Shows the internal consistency and test-retest reliability of the DCCC among students in grades 3–6 and 2, respectively.

	Internal consistency^a^	Test-retest^a^	Internal consistency^b^	Test-retest^b^
Sub1	0.817	0.647	0.848	0.685
Sub2	0.891	0.706	0.919	0.685
Sub3	0.752	0.690	0.718	0.723
Sub4	0.803	0.637	0.794	0.537
Sub5	0.763	0.615	0.749	0.719
Sub6	0.867	0.736	0.874	0.751
Sub7	0.827	0.689	0.828	0.738
Sub8	0.901	0.662	0.909	0.701
Total	0.974	0.734	0.977	0.770

*Sub1 = DCCC factor 1…Sub8 = DCCC factor 8. ^a^The reliability of DCCC among the students in grade 3 to 6, n (test-retest) = 109. ^b^The reliability of DCCC among the students in grade 2, n (test-retest) = 24.*

### Validity Statistics

Concurrent validity of the scale was assessed by calculating the correlations between the DCCC scores, the VCI score, and Chinese achievement. Consistent with cognitive theories of the DCCC, each subscale was significantly negatively associated with the ability of vocabulary comprehension and Chinese achievement among students in grades 3–6 (see **Table 4**). The same trend was detected in the sample of students in grade 2. The results supported the concurrent validity of DCCC. We also computed the correlation of eight factors to assess the relationship among factors (see **Supplementary Tables S1**, **S2**). It can be seen that all the factors were moderately associated with each other.

**Table 4:** Shows correlations among reading skill, VCI, Chinese achievement, and mathematical ability.

	VCI^a^	Chinese achievement^a^	VCI^b^	Chinese achievement^b^
Sub1	−0.229^∗^	−0.234^∗^	−0.368^∗^	−0.336^∗^
Sub2	−0.213^∗^	−0.391^∗^	−0.268^∗^	−0.449^∗^
Sub3	−0.144^∗^	−0.323^∗^	−0.226^∗^	−0.399^∗^
Sub4	−0.168^∗^	−0.297^∗^	−0.315^∗^	−0.396^∗^
Sub5	−0.125^∗^	−0.321^∗^	−0.146	−0.477^∗^
Sub6	−0.082	−0.303^∗^	−0.268^∗^	−0.498^∗^
Sub7	−0.211^∗^	−0.335^∗^	−0.261^∗^	−0.419^∗^
Sub8	−0.304^∗^	−0.385^∗^	−0.365^∗^	−0.400^∗^
Total	−0.218^∗^	−0.372^∗^	−0.324^∗^	−0.467^∗^

*Sub1 = DCCC factor 1…Sub8 = DCCC factor 8. ^a^The correlations among the measures in students grade from 3 to 6, n (VCI) = 292, n (Chinese achievement) = 454. ^b^The correlations among the measures in the students at grade two n (VCI) = 60, n (Chinese achievement) = 90. ^∗^Indicated the correlations were significant at the 0.05 level.*

## Discussion

This study was designed to test the validity and reliability of the DCCC to measure the reading ability among Chinese students in grades 2 through 6. The results revealed that reading ability could be measured in a reliable and valid manner by the parent-report scale.

Confirmatory factor analysis on the DCCC confirmed a first order factor structure among the sample of this study, which is congruent with the initial structure. The statistical indicators (χ^2^/*df* = 2.464, CFI = 0.930, TLI = 0.925, and RMSEA = 0.057) suggested a good model fit. However, the value of WRMR (1.445) was not good, and it has not been improved even after removing item 5. For the WRMR, with an increasing sample size, the likelihood of rejection of the WRMR is greater. Even with just one correlation of 0.05 misspecified in a simple model, the WRMR at a cutoff value of 1.0 rejected models over 50% of the time when *N* = 1000 (Yu, 2002). When we performed CFA in a small sample size which only included 91 students in grade 2, the WRMR was 0.991. Thus, the sample size may influence the results of the WRMR. It is acknowledged that we should take all indexes into consideration to make a decision rather than denying a model due to one bad index. The results suggested the DCCC was a valid instrument used to screen dyslexia among Chinese students in grades 2 through 6.

The 54 items of the DCCC had satisfied factor loadings that ranged from 0.383 to 0.856 on the hypothesized latent factors. Even though the remaining item 5, “enlarging the size of font or marking where you read can improve reading,” had a poor factor loading, we did not removed it because the index did not become better after removing it. Additionally, there was evidence suggesting the larger font size can help children with dyslexia to improve reading speed (Kuster et al., 2017). Thus, we believed that the DCCC would make more sense by including it.

We hypothesized that the VCI and the *Z*-scores of Chinese achievement would be negatively associated with the eight subscale scores and the total overall scores. The VCI of WISC-IV represents the cognitive ability of verbal comprehension, which is a part of literacy skills. The result indicated the students who have a higher score on the DCCC had poor performance on verbal comprehension. The students with DD have disability in reading, spelling, and comprehension. This condition causes harmful influence on reading achievement. Therefore, it is reasonable to expect a negative association between the DCCC and the *Z*-scores of Chinese achievement. Our results revealed moderate inverse associations of the score of the DCCC with Chinese achievement.

We also evaluated the reliability of the DCCC in this study. The test-retest reliability for total scale was 0.734 and ranged from 0.615 to 0.736 for eight subscales. The result showed an acceptable reliability of the DCCC. Compared to the initial study by Wu, the results showed a higher level of internal consistency estimated by Cronbach’s alpha value. The Cronbach’s alpha value is 0.974 for total scale and 0.752∼0.901 for eight subscales. The good reliability and stability indicated that the DCCC is a reliable instrument to assess the reading ability of Chinese students.

Another purpose of the present study is to evaluate the validity and reliability of the DCCC used to screen dyslexia for students in grade 2. For students in grade 2, the validity appears to be good. All subscales showed moderate relationships with the VCI and Chinese achievement, except the subscale that represented visual deficits of word recognition. All subscales also showed good reliability, except for the subscale of attention deficit. The small sample for test-retest might have accounted for the lower reliability.

Together these findings indicated that the DCCC provides a valid and reliable continuous measure of reading skills. However, this study has some limitations. First, the parent-report scale might be subjective. Considering the limitation, we used objective instruments to evaluate the validity of the DCCC. Second, the sample of this study was only recruited from Wuhan, China. This might limit the generalizability of the findings. Therefore, future research could consider assessing the validity and reliability of the DCCC on diverse samples that have a different culture and socioeconomic status in different cities of the Chinese mainland.

## Conclusion

The findings of this study indicated that the DCCC has sufficient validity and reliability. The scale proved to be able to screen Chinese dyslexia among the students in grades 2 through 6. It is significant to explore risk factors and the effective interventions for Chinese dyslexia.

## Ethics Statement

This study was carried out in accordance with the recommendations of the Ethics Committee of Tongji Medical College, Huazhong University of Science and Technology, China. The protocol was approved by the Ethics Committee of Tongji Medical College, Huazhong University of Science and Technology, China. All subjects gave written informed consent in accordance with the Declaration of Helsinki.

## Author Contributions

All authors were involved in all parts of the study and approved the final manuscript. FH, RS, and LQ designed the study. FH, HG, LL, XL, XX, and XL performed the study. FH, LQ, and JZ analyzed the data. FH, RS, and LQ wrote and reviewed the manuscript.

## Conflict of Interest Statement

The authors declare that the research was conducted in the absence of any commercial or financial relationships that could be construed as a potential conflict of interest. The reviewer AJ-C and handling Editor declared their shared affiliation.

## Abbreviations

DCCC: dyslexia checklist for Chinese children
DD: developmental dyslexia
ICC: intra class coefficient
VCI: verbal comprehension index.
