# Supplementary material for: Validity and Reliability of the Dyslexia Checklist for Chinese Children
Source: Front Psychol. 2018 Oct 9;9:1915. doi: 10.3389/fpsyg.2018.01915 (PMC6189409; doi:10.3389/fpsyg.2018.01915)
Supplement: Supplementary file 1 [file Table_1.DOCX]

| Table 1 the intercorrelations of items from Dyslexia Checklist for Chinese Children among students grade at 3 to 6 | | | | | | | |
| --- | --- | --- | --- | --- | --- | --- | --- |
|  | sub1 | sub2 | Sub3 | sub4 | sub5 | sub6 | sub7 |
| sub2 | 0.765 |  |  |  |  |  |  |
| sub3 | 0.679 | 0.762 |  |  |  |  |  |
| sub4 | 0.785 | 0.771 | 0.713 |  |  |  |  |
| sub5 | 0.592 | 0.653 | 0.618 | 0.687 |  |  |  |
| sub6 | 0.67 | 0.747 | 0.667 | 0.686 | 0.652 |  |  |
| sub7 | 0.76 | 0.779 | 0.726 | 0.822 | 0.752 | 0.704 |  |
| sub8 | 0.825 | 0.855 | 0.74 | 0.821 | 0.673 | 0.716 | 0.831 |

Sub1 = DCCC factor 1….sub8 = DCCC factor 8, n=454, All intercorrelations significant at the 0.01 level

Table 2 the intercorrelation of items from Dyslexia Checklist for Chinese Children among students in grade two

|  | sub1 | sub2 | sub3 | sub4 | sub5 | sub6 | sub7 |
| --- | --- | --- | --- | --- | --- | --- | --- |
| sub2 | 0.803 |  |  |  |  |  |  |
| sub3 | 0.668 | 0.761 |  |  |  |  |  |
| sub4 | 0.767 | 0.756 | 0.750 |  |  |  |  |
| sub5 | 0.623 | 0.644 | 0.668 | 0.721 |  |  |  |
| sub6 | 0.774 | 0.834 | 0.786 | 0.770 | 0.759 |  |  |
| sub7 | 0.758 | 0.775 | 0.727 | 0.786 | 0.765 | 0.814 |  |
| sub8 | 0.865 | 0.860 | 0.765 | 0.855 | 0.732 | 0.823 | 0.856 |

Sub1 = DCCC factor 1….sub8 = DCCC factor 8, n=91, All intercorrelations significant at the 0.01 level
